# Retraction note: Cervical type AB thymoma (mixed) tumour diagnosis in a mynah as a model to study human: clinicohistological, immunohistochemical and cytohistopathological study

**DOI:** 10.1186/s13000-016-0567-2

**Published:** 2016-11-02

**Authors:** Fariba Khaki, Javad Javanbakht, Farhang Sasani, Mohammad Javad Gharagozlou, Alimohammad Bahrami, Hemmat Moslemzadeh, Reza Sheikhzadeh

**Affiliations:** 1 Department of Pathology, Faculty of Veterinary Medicine, Tehran University, Tehran, Iran; 2 Faculty of Para-Veterinary Medicine, University of Ilam, Ilam, Iran; 3 Faculty of Veterinary Medicine, Urmia University, Urmia, Iran; 4 Faculty of Veterinary Medicine, Tehran University, Tehran, Iran

## Retraction

The Editor-in-Chief and Publisher have retracted this article [1] because the scientific integrity of the content cannot be guaranteed. An investigation by the Publisher found it to be one of a group of articles we have identified as showing evidence suggestive of attempts to subvert the peer review and publication system to inappropriately obtain or allocate authorship. This article showed evidence of plagiarism (most notably from the articles cited [2–6]) and peer review and authorship manipulation.
